# Editorial: Improving reliability of brain stimulation: What works and what doesn't?

**DOI:** 10.3389/fnhum.2023.1150586

**Published:** 2023-02-07

**Authors:** Christopher J. Gaffney, Magdalena W. Sliwinska, Gregor Thut, Helen E. Nuttall

**Affiliations:** ^1^Lancaster Medical School, Faculty of Health and Medicine, Lancaster University, Lancaster, United Kingdom; ^2^Department of Psychology, Liverpool John Moores University, Liverpool, United Kingdom; ^3^School of Psychology and Neuroscience, University of Glasgow, Glasgow, United Kingdom; ^4^Department of Psychology, Faculty of Science and Technology, Lancaster University, Lancaster, United Kingdom

**Keywords:** brain stimulation, TMS, tDCS, transcranial alternating current stimulation (tACS), TMS-EEG

## Introduction

The field of Psychology is undergoing reform following the so-called “replication crisis,” whereby a significant number of research findings have failed to replicate (Pennington, 2023). Failed replication has been associated with a lack of research transparency, questionable research practices, and a lack of methodological rigor. Psychology is a trailblazer in the Open Science movement *via* embracing preregistration and registered reports to improve overall study design and transparency of research practice, and indeed the discipline is currently undergoing significant reform and evolution. Within the disciplines of Psychology and Neuroscience, the field of brain stimulation, on which this Research Topic is focused, is not immune from issues with replication either. Brain stimulation is a non-invasive technique often used by Psychologists and Neuroscientists to manipulate brain function (Polanía et al., 2018).

Replication issues within brain stimulation research additionally reflect the high levels of inter- and intra-subject variability that is present in participants' responses to brain stimulation paradigms. One source of this variability reflects differences in the physiological state of participants. However, while Physiologists typically control for state-based differences, such as sex, time of day, menstrual cycle phase, or nutrition intake, to maximize the replicability of findings, it is not common for Psychologists and Neuroscientists to tightly control the participants' physiological state. This may be one reason for the often-unwanted variability in brain stimulation data. The source of this variability needs to be better understood, both in terms of trait-based, stable inter-subject differences (i.e., genetics), and state-based differences, which index state dependent differences between subjects (i.e., time of day).

## Interdisciplinary collaboration to improve methodological rigor

Physiologists study humans differently from Psychologists, but the two disciplines converge on measuring (neuro)physiological variables/behavioral changes underpinned by stimulation-induced changes in the brain. The purpose of this Research Topic was to entice collaboration across the fields of Physiology and Psychology to allow for cross-fertilization of scientific ideas but perhaps more importantly, experimental approach to expand understanding. This Research Topic sought to identify possible sources of variability in data collection to gather information on why experiments do or do not work out as expected, and encourage authors to highlight the strengths, but crucially the limitations of their research, so that the brain stimulation community can continue to learn and develop.

## From tDCS to TMS-EEG, TI, and tACS

Van der Cruijsen et al. investigated whether a new tDCS montage (multifocal stimulation targeting the entire motor strip) would reduce the usually high inter- and intrasubject variability in outcome of conventional tDCS. This montage has previously been reported to be superior to conventional tDCS (Fischer et al., 2017), a finding which however could not be replicated. Neither the new montage nor conventional tDCS method could increase corticospinal excitability when compared to sham treatment. Exploration of possible contributing factors to outcome variability, such as individual levels of baseline cortical excitability, did not reveal any relationship. The authors carefully consider possible accounts of their null finding and conclude that future research should include more neurophysiological measures to explore factors potentially underlying the outcome variability.

The second study by Zhang et al. examined the feasibility, safety, and blinding of temporal interference (TI) stimulation through tACS [as introduced by Grossman et al. (2017)] in a pilot study on humans. It was observed that the TI stimulation technique did not induce severe side effects or feelings of discomfort. The reported side effects were similar to those reported after receiving conventional tACS as well as to those reported in the sham groups. The blinding efficacy of TI stimulation was excellent, and there was no correlation between the severity of the side effects and the type of stimulation the participants guessed that they received. Authors acknowledge that the sample size was relatively small, which may be why the effects of TI stimulation on working memory performance were marginal. Future studies using neuroimaging techniques (e.g., functional MRI) are needed to examine the underlying neurophysiological changes in the brain regions induced by TI.

In the third TMS-EEG study with preregistration, Zazio et al. examined the robustness and functional interpretation of an early TMS-evoked potential over M1; the contralateral M1-P15. The authors replicated their previous finding that the M1-P15 is positively correlated to the ipsilateral silent period, a known peripheral measure of interhemispheric inhibition, and hence confirm that the M1-P15 is a cortical marker of callosal inhibition. In addition, their results show a modulation of this marker of transcallosal inhibition during execution of a bimanual motor task, hence strongly indicating it to be of physiological (as opposed to artifactual) nature. The study is an excellent model example how methodological rigor can improve validity of results and interpretations.

The fourth study by Roumengous et al. used paired pulse TMS to assess voluntary activation measured with transcranial magnetic stimulation (VATMS) to quantify voluntary cortical/subcortical drive to muscle. Paired pulse TMS did not modulate the biceps/triceps MEP ratio across the full range of voluntary efforts in participants with tetraplegia and did not affect the estimation of VATMS. Authors acknowledge that the sample size is small and there was a wide range of biceps strength among the participants, as well as further optimization of the protocol that is required.

To conclude, there is great opportunity in studying and understanding sources of variability that may contribute to null findings or failed replications, which could lead to much greater predictive power in brain stimulation research (Ziemann and Siebner, 2015). One way to achieve this is by maximizing methodological rigor through closer collaborations between Physiologists and Psychologists which should lead to a greater understanding of the physiological regulation of the brain (Gaffney et al., 2021), next to Open Science practice and consideration of experimental power. This presents exciting opportunities for collaboration and innovation in the future of brain stimulation research.

## Author contributions

CG and HN wrote the first draft. MS and GT critically reviewed. All authors approved the final version.
